# Case Report: A Novel CACNA1A Mutation Caused Flunarizine-Responsive Type 2 Episodic Ataxia and Hemiplegic Migraine With Abnormal MRI of Cerebral White Matter

**DOI:** 10.3389/fneur.2022.899813

**Published:** 2022-05-23

**Authors:** Xiaoqiu Yuan, Yiming Zheng, Feng Gao, Wei Sun, Zhaoxia Wang, Guiping Zhao

**Affiliations:** Department of Neurology, Peking University First Hospital, Beijing, China

**Keywords:** episodic ataxia type 2, CACNA1A, MRI, flunarizine, case report, hemiplegic migraine

## Abstract

Episodic ataxia type 2 (EA2) is one autosomal-dominant neurological disorder characterized by debilitating attacks of ataxia. It is mainly caused by loss-of-function mutations of the CACNA1A gene, which encodes the pore-forming α1A subunit of Ca_v_2.1 (P/Q type voltage-gated calcium channel). Sporadic hemiplegic migraine (SHM) is another rare disease involving CACNA1A variants, which seldom coexists with EA2. Here we report a novel pathogenic mutation in CACNA1A (c.3836dupA, exon 23, p.Y1279X) of a 16-year-old female, who complained about paroxysmal dizziness, headache, and unsteady gait. Her brain MRI revealed a slightly atrophic cerebellum and numerous asymptomatic hyperintense lesions of the cerebral white matter. The diagnosis of EA2 combined with SHM was made. Administration of 5-mg flunarizine once daily at night effectively reduced the attacks and attenuated her symptoms for a month.

## Introduction

The primary function subunit of the P/Q type voltage-gated calcium channel, commonly known as Cav2.1, is coded by CACNA1A (chromosome 19p13) (1). This complex gene contains 47 exons with abundant alternative splicing loci. Ca_v_2.1 is distributed unevenly throughout the central nervous system and is predominantly expressed in cerebellar Purkinje and granular cells. This channel primarily mediates the neurotransmitter release and regulates other crucial activities, such as cell survival (2, 3). Mutations in different sites of CACNA1A lead to various neurological disorders, collectively referred to as Ca_v_2.1 channelopathies. A wide disease spectrum has been observed, among which three classic phenotypes have been recognized, episodic ataxia type 2 (EA2), familial hemiplegic migraine type 1 (FHM1), and spinocerebellar ataxia type 6 (SCA6). Genetically, EA2 is primarily associated with loss-of-function variants of CACNA1A, whereas FHM1 is primarily associated with gain-of-function mutants, while SCA6 has an expanded CAG repeat in CACNA1A (4). Clinically, EA2 is characterized by adolescent-onset episodes of ataxia, dizziness, and nausea lasting hours to days, which can be accompanied by other cerebellar, brain stem, or cortical symptoms. SCA6 often shows a progressive cerebellar syndrome with a usual onset at middle age. FHM1 is a rare subtype of migraine characterized by aura symptoms, episodic movement weakness, migraine headaches, and a positive family history. Although most sporadic HM (SHM) cases did not show any Ca_v_2.1 mutant, it differs from FHM only in family history, suggesting the involvement of CACNA1A in SHM (5, 6). EA2 and HM can coexist within the same family (7, 8) and even in the same patient (9). The similarity of symptoms and signs makes the diagnosis of these disorders difficult, highlighting that genetic testing is necessary (4, 10). Brain MRI of EA2 patients typically reveals cerebellar atrophy or no notable findings, with cerebral white matter appearing to be rarely affected (11). However, in patients with SHM, reversible subcortical hyperintensities on images are relatively common (12). It has been reported that flunarizine is useful in treating certain CACNA1A-related disorders, such as HM, but its efficacy in EA2 remains largely unknown (9, 10). Here we describe a case of concurrent EA2 and SHM caused by a novel nonsense mutant of CACNA1A, with a slightly atrophic cerebellum, hyperintense lesions of cerebral white matter, and a favorable response to flunarizine.

## Case Presentation

A 16-year-old female visited our clinic for increasingly frequent attacks of vertigo and nausea along with gait instability. There was no related family history, such as migraines, epilepsy, or ataxia. The patient had a history of pneumonia at the age of 6 months but no history of other illnesses. She was born full-term and appeared normal in the physical, motor, social, language, and cognitive development. She is now in the third year of high school with good grades but gets relatively insufficient sleep of 5 h a day. Nevertheless, she thinks the study pressure is acceptable. The attacks could date back to a decade ago when she first experienced transient dizziness that resulted in a minor fall for no obvious reason or trigger. She recalled that she had blurred vision for a few seconds but had no other accompanying symptoms at that time. No medical attention was sought. After 2 years, the dizziness recurred leading to a distal radius fracture with no clear inducement and no other abnormalities, which was regarded as an accident, and thus neurological consultation was not provided. Episodes occurred several times at a low but increasing frequency over the next 4 years progressively. At the age of 12 years, the symptoms became non-negligible and affected her life greatly, which manifested as monthly attacks of vertigo and unsteady gaits lasting 10–20 min, accompanied by irritability, photophobia, and phonophobia. Nausea, vomiting, and slurred speech also appeared with the attacks sometimes. Between episodes, she was asymptomatic except for a slight headache. Before each episode, a moderate headache on the left side appeared for a few seconds, with no flash or other auras seen. Notable triggers included lack of sleep and strenuous exercise (such as, running 800–1,200 m). It appeared that posture and menstruation were not associated with triggering the attack. There was no tinnitus, ear fullness, diplopia, loss of consciousness, convulsions, or upper limb dysfunction during the attack. Progressively, her condition continued to deteriorate and reached 2 or 3 episodes a day, each lasting 1–2 h in the months before the visit to our clinic. In severe cases, the weakness of lower limbs or rotation of vision (according to the patient's descriptions) forced her to sit down. She once took Yangxue Qingnao granules (a type of Chinese medicine that treats headache and dizziness) and betahistine mesylate, but her condition did not improve so she interrupted the therapy. No other symptoms occurred within the 4 years before this visit. The slight headache during the interictal period had not changed.

**Figure 1:**
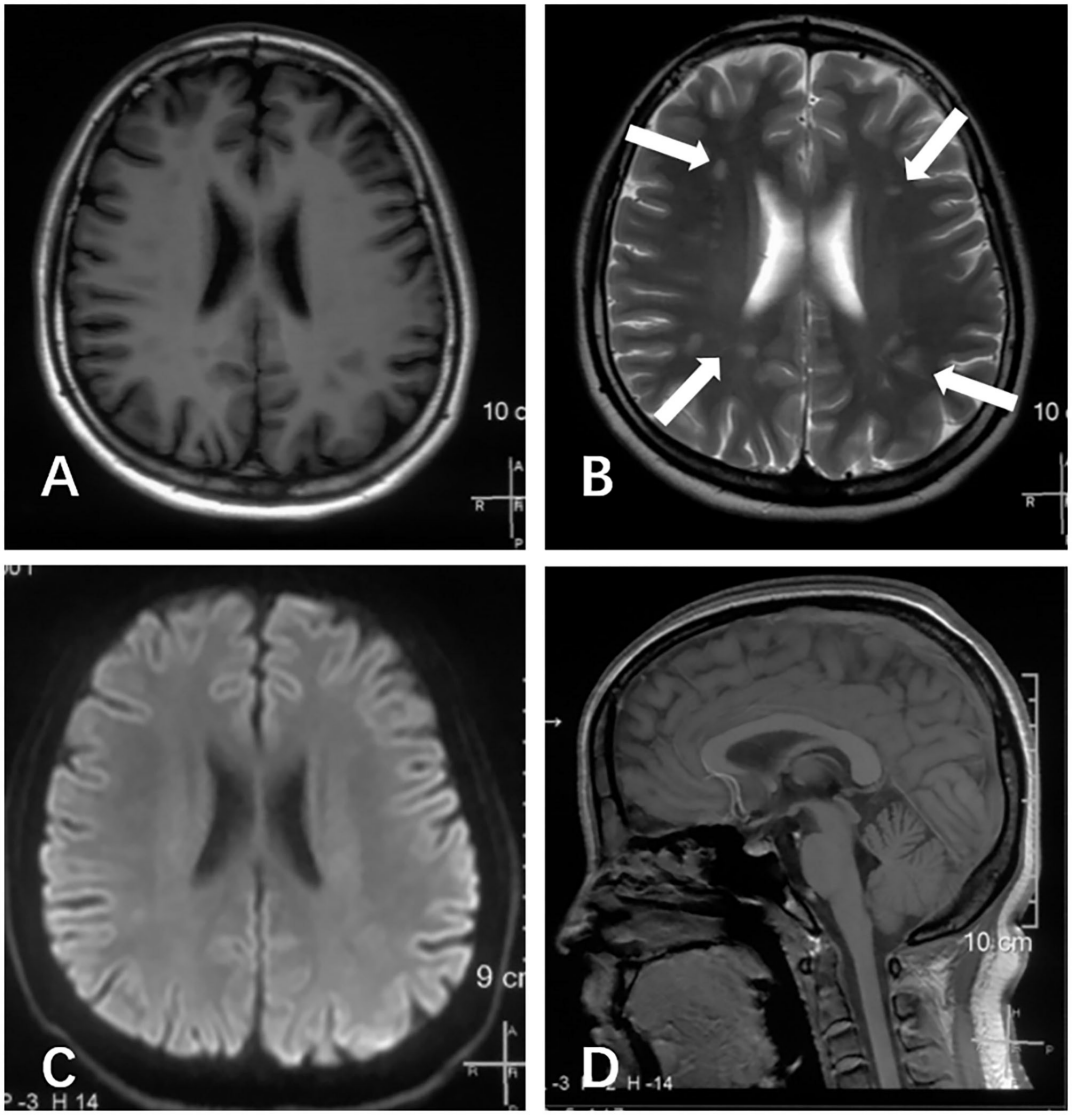
Brain MRI scans of this patient, performed at age of 16 years during the interictal period. **(A)** Axial T1 image revealed several hypointense lesions of the centrum semiovale and white matter of the bilateral frontoparietal temporal lobe. **(B)** Axial T2 image revealed several hyperintense lesions at the same locations (arrows). **(C)** The axial diffusion-weighted imaging (DWI) image appeared normal. **(D)** Sagittal T1 image demonstrated slightly cerebellum atrophy.

**Figure 2:**
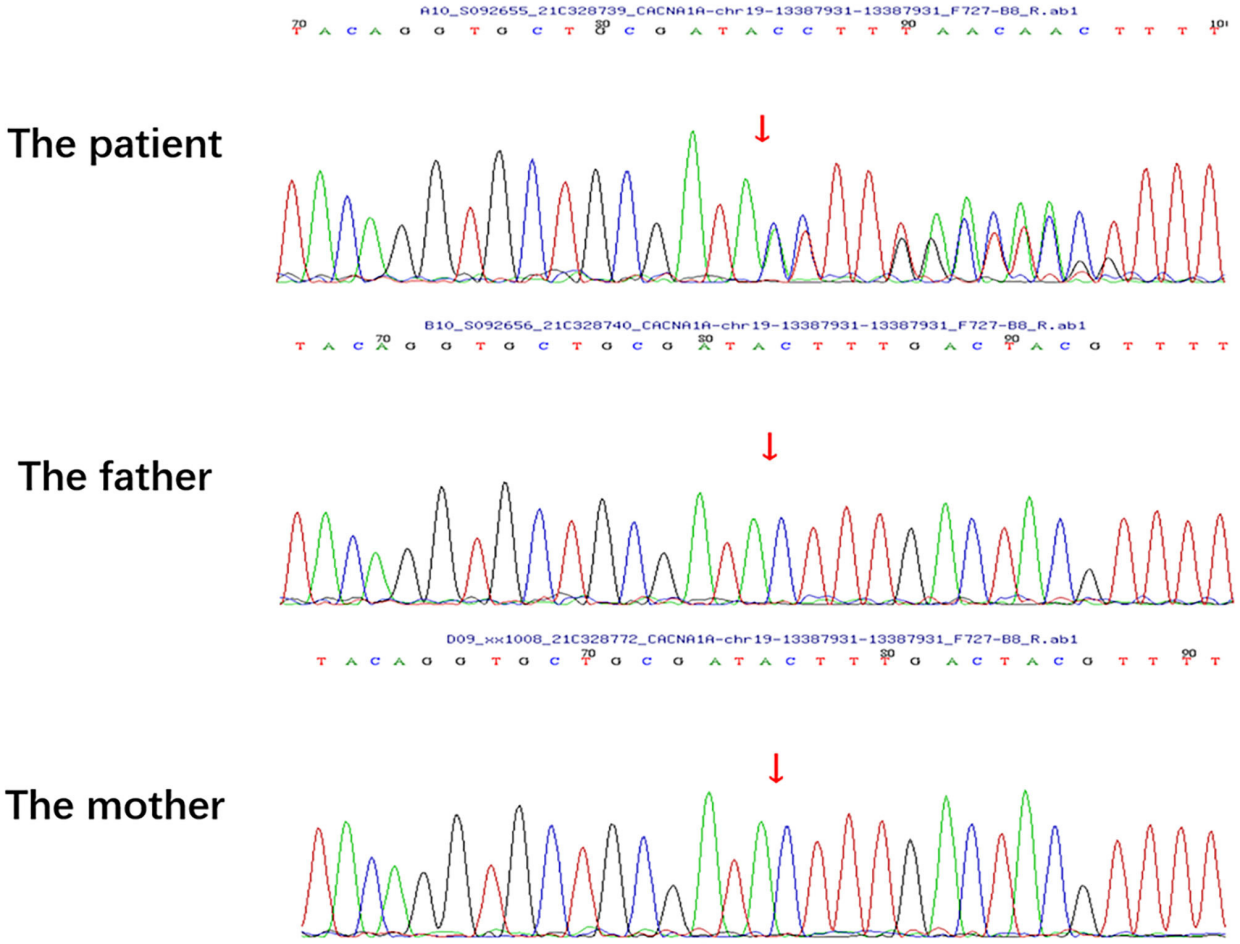
Sanger sequencing of the patient and her healthy parents. No mutation was detected in the parents at the same locus of the patient's pathogenic variant.

A physical examination was conducted during the interictal period and found no special abnormal signs of the nervous system and general condition. The patient was conscious and had fluent speech, normal memory, and normal higher cortical functioning. No nystagmus was observed. Cranial nerve tests were unremarkable. Sensations of touch, pain, temperature, vibration, and position were symmetrical and normal. The muscle strength of four limbs was five with moderate muscle tension. Tendon reflexes could be elicited symmetrically. The finger-to-nose test and heel-to-shin test were accurate. Pathological responses were negative. We failed to perform an ictal examination directly since this family lived more than 1,600 km away from our hospital and were unable to stay here for too long. We got the video recordings during an attack at home instead. The patient displayed an unsteady and broad-based gait, accurate heel-to-shin test, and absence of nystagmus (Supplementary Video). Laboratory tests of hematology, biochemistry and the homocysteine level were normal. A lumbar puncture was not performed. Magnetic resonance imaging (MRI) of the head revealed multiple patchy lesions in the centrum semiovale, and white matter of the bilateral frontoparietal temporal lobe, with T1 hypointensity (Figure 1A), T2 hyperintensity (Figure 1B), and normal in diffusion-weighted imaging (DWI) sequence (Figure 1C). The cerebellum was slightly atrophic (Figure 1D). There was no obvious change in the MRI findings compared with 1 year ago. Interictal electronystagmography showed normal recordings while electroencephalogram detected a small amount of asymmetric complex delta waves scattered in the bilateral temporal regions without other abnormal waveforms. To exclude heart and cerebrovascular diseases, we carried out electrocardiogram (ECG), echocardiography, transcranial Doppler ultrasonography, and head MRI angiography. Whole exome next-generation sequencing was conducted afterward considering the possibility of hereditary channelopathies. A novel, heterozygous mutation in the CACNA1A gene (c.3836dupA, exon 23, and p.Y1279X) leading to a premature stop codon was discovered. Further validation of Sanger sequencing for her healthy parents revealed no mutant at the same locus, indicating the identified mutation *de novo* (Figure 2). According to the recommendations of the American college of medical genetics and genomics and the association for molecular pathology, this mutation is pathogenic (PVS1 + PS2 + PM2) (13), clarifying the diagnosis of EA2 and SHM.

**Figure 3:**
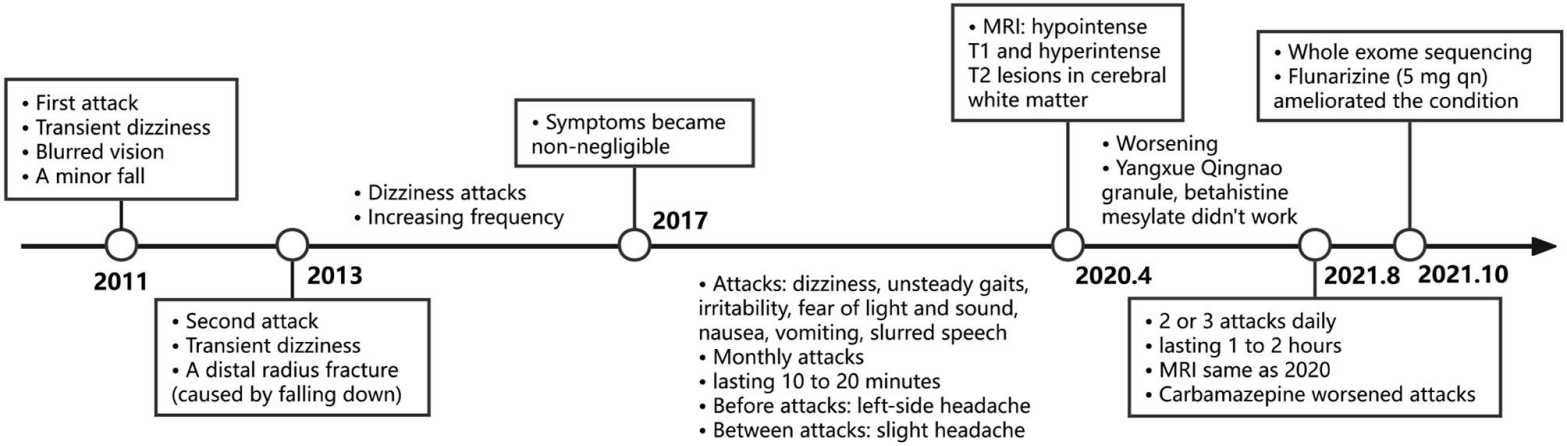
Timeline of the clinical course.

Carbamazepine (0.1 g bid) was prescribed first for symptomatic therapy before the result of the gene test but was discontinued 2 days later due to the increased attacks after initiation. She failed to start acetazolamide because this drug was unavailable in our institution or pharmacies and was hard to obtain routinely. Flunarizine of 5 mg once daily at bedtime was administered instead and effectively ameliorated her condition in a month. According to her description, the frequency decreased from 2 or 3 episodes a day to once a month, the attack duration declined from 1 or 2 h to 30 min, interictal and ictal headache almost disappeared, and the symptoms of episodes of dizziness, nausea, and gait instability were significantly relieved. Our patient has not taken triptans or other pain-killing medications because the headache she felt was not that disturbing. The timeline of the clinical course is presented in Figure 3.

## Discussion

Our patient had the typical manifestations of episodic dizziness and ataxia accompanied by nausea, vomiting, and slurred speech. Her MRI presentation was distinctive but lacked specificity (discussed later), while other examinations appeared normal. Next-generation sequencing revealed one truncating mutant of CACNA1A and pointed to the diagnosis of EA2 at first. However, some symptoms atypical in EA2 but common in SHM, such as irritability, the fleeting unilateral headache just before episodes, as well as the photophobia, and phonophobia during an attack, led us to consider the coexistence of SHM. This supposition was further supported by her adequate response to the classic migraine prevention drug flunarizine and the hyperintense cerebral white matter, which is known in patients with migraine. However, the headaches coming before other symptoms and lasting for a very short time during an attack seemed not to meet the diagnostic criteria ICHD3 (6). Clear identification of CACNA1A-related diseases could be difficult even after getting the gene results. Huge variations and some degree of overlaps have been observed in the presentations of EA2 and HM cases (7, 14). EA2 and HM symptoms did occur in different individuals with the same CACNA1A mutant (7, 8) and even in a single patient (9). The concurrence of these two diseases might cause confusing episodes of ataxia, dizziness, and headaches in our patient. Notably, her manifestations also overlapped immensely with vestibular migraine (VM), which is the most common cause of episodic vertigo and is also characterized by headache, photophobia, and phonophobia. Differentiating EA2 from this disease based on clinical presentation alone could be quite confusing. According to the ICHD3 criteria, VM is a diagnosis of exclusion (15). EA2 and SHM seemed to be a better explanation here given the signature gene CACNA1A. Nonetheless, VM cannot be completely ruled out. The exclusive procedures in previous studies might prevent the identification of candidate genes since VM would be left out whenever a proven gene related to another disease was detected. CACNA1A could be one of the pathogenic genes of VM or not. Additionally, the young age of our patient and her satisfactory response to flunarizine were suspicious. Some children experiencing benign paroxysmal vertigo later develop VM (16). Long-term follow-up is required.

The majority of reported EA2 cases were caused by truncating mutations of CACNA1A, while the pathogenic role of this gene in SHM1 is not that clear (6, 11). Here, the new nonsense mutation in exon 23 of CACNA1A forces translation to terminate at the domain III of Ca_v_2.1 (2, 3), leading to incomplete channels with probably weakened conductance of Ca^2+^ (17). Neurotransmission in the cerebellum and the pace-making of Purkinje cells could get impaired given their dependency on Ca_v_2.1 (1, 2, 4). Moreover, the lower inflow through Ca_v_2.1 eminently stimulates other Ca^2+^ currents into neurons, particularly the L type (17). The overall Ca^2+^ influx increases, as a result, engendering further cytotoxicity, excessive glutamate in the synaptic gap, and the increased susceptibility to cortical spreading depression (CSD) (5, 18). Interestingly, the C-terminus is cleaved from the full-length α_1A_ subunit followed by entry into the nucleus. This translocation process appears uniquely vital for the survival of Purkinje cells but is absent in the case of a truncated protein with no C-terminus, resulting in the deterioration of cerebellar neurons (2, 3). Nonetheless, the above theories are far from adequately explaining the clinical manifestations. The mechanisms underlying Ca_v_2.1 channelopathies remain poorly understood at present.

Our patient displayed a slightly atrophic cerebellum on images, consistent with the MRI of most CACNA1A-mutant patients, which partially resulted from the degeneration and death of Purkinje cells mentioned above. Abnormal signals of cerebral white matter exhibited in our case have not been described in EA2 but have occasionally appeared in SHM cases with or without CACNA1A mutations (12). Hyperintense lesions on T2 have also been observed in other Ca_v_2.1 channelopathies, including an infant boy with severe encephalopathy (19) and a family with FHM (20). We suspected that the lesions showed in our patient were caused by microangiopathy-related demyelination, considering the patchy locations and the modes of hypointense T1 and hyperintense T2 (21). Possible pathological evidence came from the ultrastructural examination of biopsy samples in the above-mentioned FHM family, which revealed microangiopathy in skin and muscle (20). Oligodendrocyte progenitor cells, responsible for myelin-forming, were reported to selectively deteriorate in the corpus callosum when Ca_v_1.2 was deleted, implying the essential involvement of voltage-gated calcium channels in myelination (22). Intriguingly, nerve bundles from the corpus callosum are the main component of the centrum semiovale, one of the abnormal regions of our patient. In models of brain injury, the loss-of-function mutant Ca_v_2.1 demonstrated suppression of astrocyte activation, which is involved in remyelination failure (23). Thus, we proposed that the myelin loss derived from Ca_v_2.1-defect-caused microangiopathy brings about the aforementioned abnormal signals. CSD in SHM, which activates the release of vasoactive neuropeptides and triggers inflammation may also account for the image (5). Nevertheless, the relationship between lesions of superficial white matter and Ca_v_1.2 as one L-type channel is still unknown. We cannot rule out the possibility that these abnormal signals have nothing to do with EA2, given no change in MRI over the year while the condition worsened, the absence of any symptoms related to white matter abnormalities, and the lack of earlier images. Further observations in clinical practice are needed.

The majority of patients with Ca_v_2.1 channelopathy underwent progressively worsening conditions without proper intervention and were severely affected by the attacks (4), indicating the importance of prompt detection, correct diagnosis, and use of appropriate drugs. Acetazolamide is recommended to treat EA2 and SCA6 for relieving symptoms and slowing disease deterioration; 4-aminopyridine, a potassium channel blocker inhibiting Ca^2+^ inflow indirectly, has shown favorable effects in some EA2 cases (4, 11). However, in our case, neither was readily available while flunarizine was accessible and much cheaper. Flunarizine protects the neurons from ionic overload efficiently by inhibiting the L-type calcium and voltage-gated sodium channels (18). Recent guidelines for HM suggest oral flunarizine at 10 mg/day but this recommendation was largely based on studies in adults with no focus on CACNA1A-caused HM (5, 6). Instead, previous CACNA1A-related cases showing the efficacy of flunarizine were both at the daily dose of 5 mg, involving one FHM1 family and noteworthily, a woman suffering from episodic ataxia and HM (9, 10). A study summarizing 11 years' experience in treating childhood migraine found flunarizine effective in HM at an initial dose of 5 mg daily. Escalation to 7.5 or 10 mg/day took place only when there was an insufficient response to the starting dose (24). It is worth noting that flunarizine may act differently in patients with the same mutation, and several side effects (such as, drowsiness, weight gain, depressive, or extrapyramidal syndromes) could arise in long-term applications (9). Follow-up for the possible progression of cerebellar atrophy and timely treatment modification are needed. Diagnosis of Ca_v_2.1 channelopathies is largely facilitated by molecular approaches. Treatments with higher efficacy and fewer side effects demand further investigations. Rehabilitation of impaired neurologic functions and genetic counseling for the families of such patients also represent topics that should not be ignored in the future.

In conclusion, Ca_v_2.1 channelopathies should be considered when a teenager presents with episodic dizziness, unsteady gait, and headache. The possibility of pluralism should be assessed when a single phenotype cannot display all clinical manifestations. Patients with CACNA1A-related diseases could display cerebellum atrophy or multiple patchy abnormal signals of cerebral white matter on MRI. Flunarizine could serve as one choice in such cases. More studies are needed to confirm the efficacy and possible adverse effects of long-term use. Continuing follow-up is necessary to timely stop progressive damage to the central nervous system.

## Data Availability Statement

The datasets presented in this article are not readily available because of ethical and privacy restrictions. Requests to access the datasets should be directed to the corresponding author.

## Ethics Statement

The studies involving human participants were reviewed and approved by Institutional Review Board and Ethics Committee at Peking University First Hospital. Written informed consent to participate in this study was provided by the participants' legal guardian/next of kin. Written informed consent was obtained from the minor(s)' legal guardian/next of kin for the publication of any potentially identifiable images or data included in this article.

## Author Contributions

XY prepared the original draft. YZ contributed with the acquisition of data and revising the manuscript. FG, WS, and ZW revised the manuscript. GZ reviewed and edited the final manuscript. All authors contributed to the article and approved the submitted version.

## Funding

This study was supported by the Scientific Research Seed Fund of Peking University First Hospital (2018SF033). Funding bodies did not play a role in the collection, analysis, and interpretation of data. Funding bodies did not contribute to the writing of this manuscript.

## Conflict of Interest

The authors declare that the research was conducted in the absence of any commercial or financial relationships that could be construed as a potential conflict of interest.

## Publisher's Note

All claims expressed in this article are solely those of the authors and do not necessarily represent those of their affiliated organizations, or those of the publisher, the editors and the reviewers. Any product that may be evaluated in this article, or claim that may be made by its manufacturer, is not guaranteed or endorsed by the publisher.
