# Supplementary material for: Case Report: A Novel CACNA1A Mutation Caused Flunarizine-Responsive Type 2 Episodic Ataxia and Hemiplegic Migraine With Abnormal MRI of Cerebral White Matter
Source: Front Neurol. 2022 May 23;13:899813. doi: 10.3389/fneur.2022.899813 (PMC9168224; doi:10.3389/fneur.2022.899813)
Supplement: Supplementary file 1 [file Table_1.DOCX]

Supplementary Material

**Supplementary Video** The patient showed unsteady and broad-based gait, accurate heel-to-shin test, and the absence of nystagmus during an attack at home.
